# Principles of fluorescence correlation spectroscopy applied to studies of biomolecular liquid–liquid phase separation

**DOI:** 10.52601/bpr.2022.210047

**Published:** 2022-04-30

**Authors:** Zhulou Wang, Huizhi Zhang, Lin Jian, Bo Ding, Keying Huang, Wolun Zhang, Qian Xiao, Shaohui Huang

**Affiliations:** 1 Institute of Biophysics, Chinese Academy of Sciences, Beijing 100101, China; 2 School of Life Sciences, University of Chinese Academy of Sciences, Beijing 101499, China; 3 LightEdge Technologies Limited, Zhongshan, Guangdong 528403, China

**Keywords:** Liquid–liquid phase separation, Fluorescence correlation spectroscopy, Diffusion coefficient, Hydrodynamic radius, Binding affinity

## Abstract

Fluorescence correlation spectroscopy (FCS) investigates the temporal relationship of fluctuating fluorescence signals reflecting underlying molecular processes occurring in a solution sample or a single live cell. This review article introduces the principles of two basic and most used FCS techniques: fluorescence auto-correlation spectroscopy (FACS) and fluorescence cross-correlation spectroscopy (FCCS). Combined, FACS and FCCS techniques can quantitatively analyze multiple properties of molecule or nanoparticle samples, including molar concentration, diffusion coefficient and hydrodynamic radius, homo- or hetero-interaction, fluorescence brightness, *etc*. Not surprisingly, FCS techniques have long been used to investigate molecular mechanisms of biomolecular phase separation, first in the lipid bilayer and more recently in cell cytosol and nucleoplasm. The latter applications are especially exciting since a whole new class of membraneless cellular organelles have been discovered, which are proposed to be results of biomolecule liquid-liquid phase separation (LLPS). LLPS research can benefit significantly from the multifunctionality and single-molecule sensitivity of a variety of FCS techniques, particularly for live-cell studies. This review illustrates how FACS and FCCS techniques can be used to investigate multiple aspects of the molecular mechanisms of LLPS, and summarizes FCS applications to LLPS research *in vivo* and *in vitro*.

## INTRODUCTION TO FLUORESCENCE CORRELATION SPECTROSCOPY

Traditional fluorescence spectroscopy investigates photophysical properties of fluorescence photons, such as excitation and emission spectra, polarization, lifetime, quenching, energy transfer, *etc*. In contrast, fluorescence correlation spectroscopy (FCS) and image correlation spectroscopy (ICS) analyze relationships (*i.e.*, correlation) of fluorescence signals in temporal and spatial domains, respectively. This review focuses on the FCS techniques. Excellent reviews regarding the ICS techniques can be found elsewhere (Hebert* et al.* 2005; Kolin and Wiseman 2007; Petersen* et al.* 1993). Temporal relationships of fluorescence signals are caused by underlying physical (*e.g.*, diffusion) and/or chemical (*e.g.*, binding) molecular processes. Thus, FCS can be used to investigate physical (*e.g.*, diffusional coefficient, hydrodynamic radius) and chemical (*e.g.*, concentration, binding affinity) properties of fluorescent or fluorescently labeled molecules. The theoretical foundation of FCS was established in the early 1970s (Ehrenberg and Rigler 1974; Elson and Magde 1974; Magde* et al.* 1972, 1974), and its mathematic formulation is similar to that of dynamic light scattering (DLS). DLS utilizes the nonspecific and much weaker signal of scattered photons, thus requiring purified and concentrated sample solutions. Using specific and highly amplified fluorescent photons, FCS has the ultimate sensitivity (*i.e.*, single molecule) and can investigate molecular properties in a heterogeneous environment (*e.g.*, within a single live cell). In fact, FCS is the first single-molecule fluorescence technique (Eigen and Rigler 1994). Widespread applications of FCS methods only began in the early 1990s, after the development and maturation of laser, detector, computer, microscope, fluorescence label, data acquisition and analysis technologies. Today, there have been >12,000 papers in PubMed published using a variety of FCS techniques, covering research fields of biology, medicine, chemistry, physics, nanotechnology, *etc*. FCS has been proved to be a versatile tool for investigating molecular properties in aqueous solutions or in single live cells. Through auto- or cross-correlation analysis of temporally fluctuating fluorescence signals collected from an ultra-small open volume (~1 femtoliter; fL), FCS can quantify molecular properties such as concentration (pmol/L–nmol/L), size (hydrodynamic radius) and interaction (*K*_D_ value) with single-molecule resolution (Elson 2011; Haustein and Schwille 2003; Hess* et al.* 2002; Wang* et al.* 2018). Particularly, FCS has long been used to investigate phase separation processes in the lipid bilayer (Chiantia* et al.* 2009; Eggeling* et al.* 2009; He and Marguet 2011; Korlach* et al.* 1999; Mueller* et al.* 2013). Recently, FCS has been applied to studies of biomolecular liquid–liquid phase separation (LLPS) *in vivo* or *in vitro*. This review will first explain, at a basic level, principles of FCS analysis, and then summarize FCS applications to LLPS studies in live cells and in aqueous solutions.

## PRINCIPLES OF FLUORESCENCE CORRELATION SPECTROSCOPY

The most updated and comprehensive explanation of the FCS techniques can be found in a recently published book entitled “An Introduction to Fluorescence Correlation Spectroscopy” by IOP Publishing (Bristol, UK) (Wohland* et al.* 2020). Interested readers are strongly recommended to study this book for an in-depth understanding of a variety of fluorescence correlation methods, their capabilities, applications and pitfalls. Below, we introduce the two basic and most widely used FCS techniques: fluorescence auto-correlation spectroscopy (FACS; often just referred to as FCS) and fluorescence cross-correlation spectroscopy (FCCS). These two techniques analyze quantitatively these fundamental molecular properties: molar concentration, diffusion coefficient and hydrodynamic radius, hetero- and homo-interactions. In addition, the brightness of fluorescently labeled molecules can also be estimated experimentally. These analytical capabilities, utilized separately or in combination, provide powerful means of investigating molecular mechanisms of LLPS, in live cells or in aqueous solutions. We attempt to explain the principles of FCS analysis both in intuitive and quantitative ways.

The most distinguishing feature of FCS techniques is to investigate molecular properties in an equilibrium solution. Typical approaches to investigate chemical reactions using the “perturbation-monitoring” method: *e.g.*, adding reactants A and B together at time zero (*i.e.*, experiment starts), then monitoring the formation of the binding product AB until the reaction reaches equilibrium:

$ \left[A\right]+\left[B\right]{\rightarrow}\left[AB\right] . $

Here, the association rate constant *k*_f_ is much larger than the dissociation rate constant *k*_b_, and brackets represent molar concentrations. In contrast, FCS techniques often start with a sample solution already in equilibrium! In this case, one of the reactants is labeled with a fluorescent probe (A^*^), and the chemical reaction either causes A^*^ to diffuse slower (by forming the A^*^ B product), and/or results in stronger or weaker A^*^ fluorescence:

$ [A^{ *}]+\left[B\right]\leftrightarrow \left[{A}^{ *}B\right]. $

Obviously, for a bulk solution in chemical equilibrium, the amount of AB formation equals that of its dissociation. Thus, the total fluorescence of the bulk sample remains constant and yields no information about the underlying reaction kinetics. However, if we can isolate a microscopic part of the bulk sample, within which chemical reaction occurs with single or a few molecules, then the association and dissociation rates are stochastically determined by individual thermodynamic energies associated with these molecules, causing instant unbalancing of the chemical reaction:

$ \left[{A}^{ *}\right]+\left[B\right]{\rightarrow}\left[{A}^{ *}B\right]\;{\rm{or}}\;\left[{A}^{ *}\right]+\left[B\right]{\leftarrow}\left[{A}^{ *}B\right] . $

Such instant perturbations are reflected by fluorescence fluctuations observed from the microscopic part of the sample. Auto- or cross-correlation analysis of the fluctuating fluorescence signals can then uncover the underlying rate constants associated with the chemical reaction.

**Figure 1:**
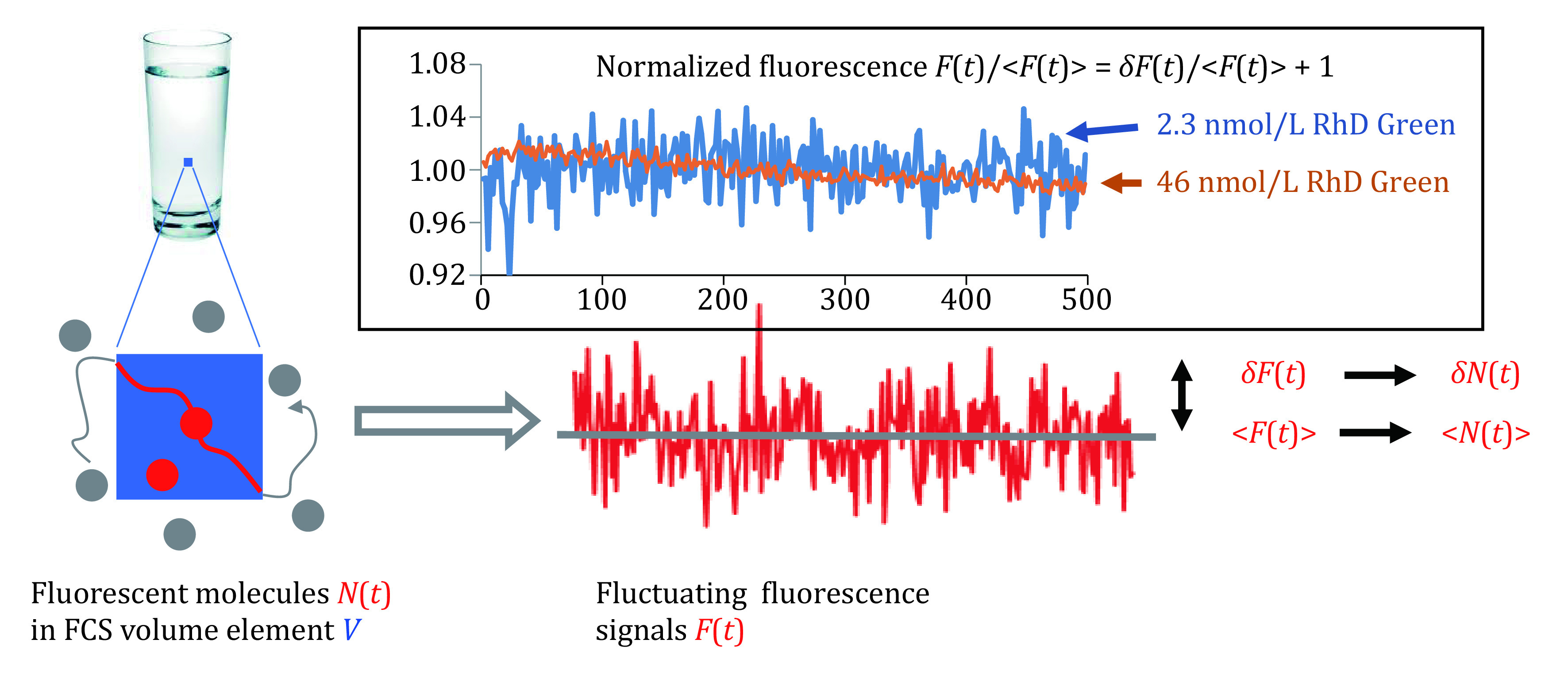
Correlation between fluctuating fluorescence signals and numbers of freely diffusing fluorescent molecules within a microscopic volume (*i.e.*, FCS volume element) of a bulk sample solution. Inset shows normalized fluorescence intensities of two Rhodamine Green samples obtained from such an FCS volume element

For FCS techniques, this microscopic part of the bulk solution is isolated using a focused laser beam created by a microscope optical pathway. We call this microscopic part as “FCS volume element V” (Fig. 1). *V* is very small, typically less than one femtoliter (1 fL = 10^−15^ L), within which single or few (<100) non-interacting fluorescent molecules of the same species diffuse freely. *V* is also an open volume in a sample solution, so fluorescently labeled molecules can diffuse in and out of it. At any moment *t*, there are *N*(*t*) number of fluorescent molecules within *V*. *N*(*t*) is stochastically determined by the few freely diffusing molecules sampled by the FCS volume element *V* at time *t*. In fact, *N*(*t*) obeys Poisson statistics, thus its time averaged (represented by angle brackets) value <*N*(*t*)> equals its variance <*δN*(*t*)^2^>:

1 $ \mathrm{P}\left[N\left(t\right)\right]=\frac{{ < N\left(t\right) > }^{N\left(t\right)}{e}^{- < N\left(t\right) > }}{N\left(t\right)!}\to  < N\left(t\right) > \mathrm{ }=\mathrm{ } < {\delta N\left(t\right)}^{2} > . $

In Eq. 1, *δN*(*t*) = *N*(*t*) – <*N*(*t*)>, indicating instant deviations of the numbers of fluorescent molecules within *V* from the time averaged value. For a single fluorescent species of equal molecular brightness, the average number of fluorescent molecules <*N*(*t*)> and its instant fluctuations *δN*(*t*) are reflected respectively by the average fluorescence intensity <*F*(*t*)> and its intensity fluctuations *δF*(*t*) (Fig. 1). Thus,

2 $ < F\left(t\right) >  = < {\delta F\left(t\right)}^{2} > .$

In Eq. 2, *δF*(*t*) = *F*(*t*) – <*F*(*t*)>. As mentioned above, auto-correlation analysis of the fluctuating fluorescence signal *F*(*t*) can uncover underlying molecular properties. The auto-correlation function is defined as:

3 $ G\left(\tau \right)=\frac{ < F\left(t\right)F\left(t+\tau \right) > }{{ < F\left(t\right) > }^{2}}=\frac{ < \delta F\left(t\right)\delta F\left(t+\tau \right) > }{{ < F\left(t\right) > }^{2}}+1. $

In Eq. 3, *G*(*τ*) is the auto-correlation amplitude and *τ* is the correlation time. Setting *τ* = 0 and substituting Eq. 2, Eq. 3 becomes:

4 $ G\left(0\right)=\frac{ < {\delta F\left(t\right)}^{2} > }{{ < F\left(t\right) > }^{2}}+1=\frac{1}{ < F\left(t\right) > }+1. $

Equation 4 indicates that the normalized fluorescence fluctuation amplitude <*δF*(*t*)^2^>/<*F*(*t*)>^2^ is inversely proportional to the average fluorescence intensity <*F*(*t*)>. This inverse relationship can be experimentally verified (inset in Fig. 1): *i.e.*, under the same experimental conditions, the average fluorescence intensity of a 2.3 nmol/L Rhodamine (RhD) Green sample is obviously much weaker than that of a 46 nmol/L RhD Green sample; yet, the normalized fluorescence fluctuation amplitudes of the former are much bigger than those of the latter. Equation 4 can be rewritten using the number of fluorescent molecules *N*(*t*) within *V* and its instant fluctuation *δN*(*t*):

5 $ G\left(0\right)=\frac{ < {\delta N\left(t\right)}^{2} > }{{ < N\left(t\right) > }^{2}}+1=\frac{1}{ < N\left(t\right) > }+1=\frac{1}{CV{N}_{\mathrm{A}}}+1.$

In Eq. 5, *C* is the molar concentration of a sample solution and *N*_A_ is the Avogadro constant. Combined, Eqs. 4 and 5 indicate that auto-correlation analysis of a fluctuating fluorescence signal *F*(*t*) can determine the *G*(0) value (Eq. 4), from which molar concentration can be calculated (Eq. 5). Of course, we also need to determine the FCS volume element *V*, which is typically done using a 1–10 nmol/L calibration sample containing a fluorescent dye (*e.g.*, Alexa488, ATTO655) with a known concentration or diffusion coefficient (see below).

Equation 4 seems to suggest that the *G*(0) value can be directly calculated through inversion of the average fluorescence intensity (*i.e.*, 1/<*F*(*t*)>). In reality, the experimentally detected <*F*(*t*)> contains not only fluorescence photons originated from samples molecules in *V*, but also background signals (*e.g.*, solvent scattered light, intrinsic fluorescence), detector noises (*e.g.*, dark current, after pulse), *etc*. Thus, the correct *G*(0) value is determined indirectly through quantitative evaluation of the auto-correlation function over a wider range of correlation times (*i.e.*, 0 < *τ* < ∞), as explained below.

Equation 5 indicates that there shall not be too many fluorescent molecules within the FCS volume element *V*. For example, if there are more than 1000 molecules in *V*, *G*(0) will be less than 0.001, then the normalized fluorescence fluctuation amplitudes will be too small to be instrumentally evaluated (Eq. 4). If *V* = 1 fL, then 1000 molecules within it are equivalent to a molar concentration of 1.66 μmol/L. Biomolecule concentrations in live cells are typically in the μmol/L range, thus we shall make *V* as small as possible so that FCS techniques can be applied to live-cell studies. The smallest 3D-volume, from which fluorescence signals are collected, actually defines a fluorescence microscope’s axial and lateral resolutions. Thus, pursuing the smallest signal collection volume is the unified goal of FCS and fluorescence microscopy. The spatial resolution of conventional fluorescence microscopy is principally determined by the numeric aperture (NA) of a microscope objective as well as the wavelength of fluorescence emission. Furthermore, FCS typically investigates molecule diffusion in an aqueous solution. Thus, a water-immersion, high-NA objective with correction collar for compensating glass coverslip thickness (*e.g.*, Olympus 60X 1.2NA water-immersion objective) is usually employed for FCS experiments. FCS analysis has been adapted to most high-resolution fluorescence microscopes, including confocal, multiphoton, TIRF, light-sheet, *etc*. In addition, FCS is compatible with stimulated emission depletion (STED) super-resolution fluorescence microscopy (Honigmann *et al*. 2014; Mueller *et al*. 2013). A much smaller FCS volume element defined by STED microscopy allows fluorescent samples with higher molecular concentrations (Eq. 5). More importantly, a smaller FCS probing volume enables precise investigation of biologically important structures such as “lipid rafts” that are smaller than the spatial resolution of conventional fluorescence microscopy (Honigmann *et al*. 2014; Mueller *et al*. 2013). Overall, a combination of fluorescence imaging and correlation analysis enables not only visualization of subcellular structures in a live cell, but also the investigation of molecular mechanisms on the subcellular structures!

**Figure 2:**
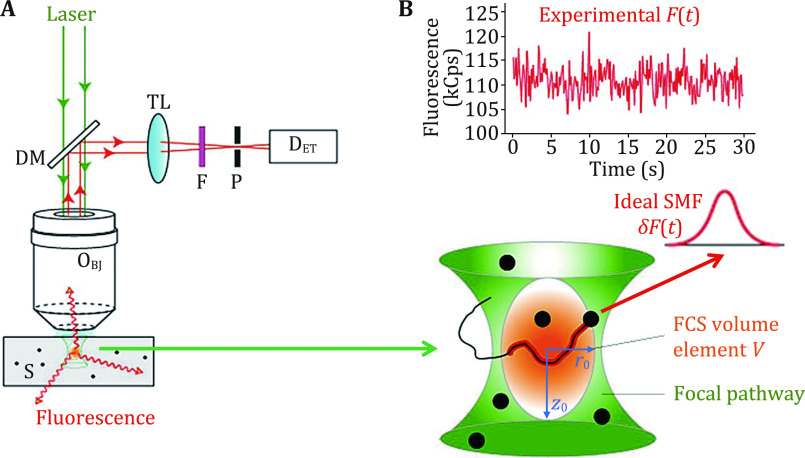
Fluorescence signals collected from an FCS volume element created through a confocal optical pathway. **A** Confocal optical pathway. **B** Bottom: FCS volume element and an ideal single molecule fluorescence signal; top: real fluorescence signals collected from the FCS volume element

Figure 2A shows an FCS device built upon a confocal optical pathway. The laser beam is focused into a sample solution via a high numeric aperture, water-immersion objective. Fluorescent molecules within the focal pathway are excited and resulting fluorescence signals are partially collected by the same objective, reflected by a dichroic mirror, cleaned up by a filter before being detected by a single-photon counting avalanche photodiode (SPAD). The key for confocal microscopy is to place a confocal pinhole of suitable size and position in front of the SPAD. The function of this pinhole is to further restrict a fluorescence signal collection volume (*i.e.*, FCS volume element *V*) within the focal spot in the sample solution (Fig. 2B, bottom). Within the elliptically shaped volume element, the laser intensity is highest at the center (*I*_0_) and decays approximately in Gaussian modes both in the axial and lateral directions. The boundaries of *V* are defined at *r*_0_ and *z*_0_, where laser intensities decay to 1/e^2^ of *I*_0_ along with lateral and axial directions respectively. When a single fluorescently labeled molecule diffuses through *V*, its fluorescence intensity along the diffusing pathway will match laser intensities along the pathway. In an ideal experiment, when a single molecule diffuses straight through the center of the FCS volume element in the lateral direction, it will produce an ideal single-molecule fluorescence (SMF) signal *δF*(*t*) that ignores background signals and detector noises. *δF*(*t*) has a Gaussian shape and its width reflects the time for this molecule to diffuse through *V* (Fig. 2B, bottom). Obviously, a big molecule with a smaller diffusion coefficient will produce a wider *δF*(*t*), and a small molecule with a narrower *δF*(*t*). Thus, SMF signals acquired from the FCS volume element contain information about molecular size! The experimentally acquired fluorescence signal *F*(*t*) is a linear combination of a limited number of SMF signals at any moment *t* (Fig. 2, top). These SMF signals do not have the ideal Gaussian shape, but nevertheless still contain information about molecule size. Auto-correlation analysis of the experimentally collected *F*(*t*) will yield quantitative results about a sample molecule’s diffusion coefficient as well as its hydrodynamic radius.

**Figure 3:**
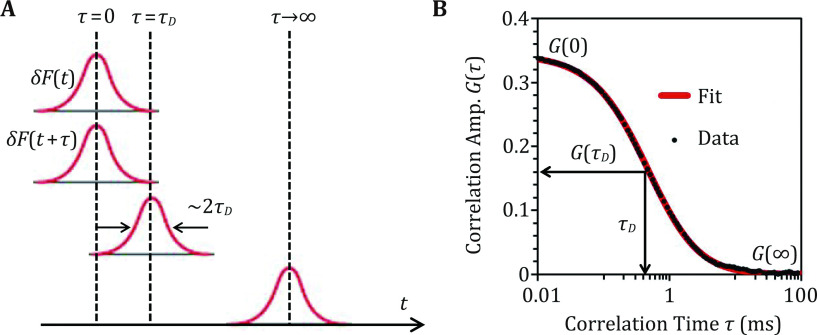
Pictorial explanation of fluorescence auto-correlation analysis. **A** Auto-correlation of an ideal single molecule fluorescence signal. **B** Corresponding auto-correlation decay curve of the ideal single molecule fluorescence signal

Within the framework of the ideal SMF experiment discussed above. The auto-correlation function (Eq. 3) can be visualized as finding the overlapping area between the original SMF signal *δF*(*t*) and its replicate signal *δF*(*t* + *τ*), with *δF*(*t* + *τ*) being shifted on the horizontal time axis by a *τ* unit (Fig. 3A). When *τ* = 0, *δF*(*t*) and *δF*(*t* + *τ*) completely overlap, thus producing the biggest auto-correlation amplitude *G*(0) (Fig. 3B). When *τ* = *τ*_*D*_, approximately half of the *δF*(*t*) and *δF*(*t* + *τ*) areas overlap, producing a *G*(*τ*_*D*_) amplitude that is roughly half of the *G*(0) amplitude. The characteristic diffusion correlation time *τ*_*D*_ indicates how fast the auto-correlation function *G*(*τ*) decays. In our ideal SMF experiment, *δF*(*t*) width is ~2*τ*_*D*_; thus, *τ*_*D*_ is related to molecular size! When *τ* → ∞, *δF*(*t*) and *δF*(*t* + *τ*) completely separate on the time axis, thus *G*(∞) → 0. Overall, *τ* increase results in *G*(*τ*) decrease, producing an auto-correlation decay curve shown in Fig. 3B. From the *G*(0) value of the decay curve, we can derive the molar concentration of a sample solution (Eq. 5), and from the *τ*_*D*_ value, we can calculate the diffusion coefficient and hydrodynamic radius of the sample molecule (see below). Importantly, our pictorial explanation of the auto-correlation analysis can be extended to the experimentally acquired fluorescence signal *F*(*t*) (Fig. 2B, top). Essentially, the auto-correlation function (Eq. 3) is a mathematic funnel: *i.e.*, inputting the fluctuating fluorescence signals *F*(*t*), outputting the auto-correlation decay curve; from the latter molar concentration and diffusion coefficient of a fluorescent molecule can be precisely measured.

Auto-correlation computation can be accomplished using a hardware correlator (Eid* et al.* 2000; Gamari* et al.* 2014) or through software algorithms (Magatti and Ferri 2003; Schaub 2013), producing discrete *G*(*τ*) data shown in Fig. 3B. Quantitative interpretation of the *G*(*τ*) data requires the mathematic modeling of three-dimensional (3D) molecular diffusion sampled by the FCS volume element *V* (Fig. 2B, bottom). Assuming a uniform solution containing one species of non-interacting fluorescent molecules, the analytical solution to the auto-correlation function, in terms of 3D-molecular diffusion, is (Elson and Magde 1974; Krichevsky and Bonnet 2002):

6 $ G\left(\tau \right)=\frac{1}{N}\cdot \frac{1}{\left(1+\dfrac{\tau }{{\tau }_{D}}\right)\sqrt{1+\dfrac{\tau }{{\tau }_{D}{S}^{2}}}},\;G\left(0\right)=\frac{1}{N}\;\;. $

In Eq. 6, *N* = <*N*(*t*)>, and *S* describes the ellipticity of the FCS volume element *V* (Fig. 2B, bottom), with *S* = *z*_0_/*r*_0_ (Fig. 2B, bottom). Using Eq. 6 and setting *N*, *τ*_*D*_ and *S* as fitting parameters, raw *G*(*τ*) data (black dots in Fig. 3B) can be quantitatively analyzed via a nonlinear least squared fitting algorithm (red line in Fig. 3B), producing experimentally determined values of *N*, *τ*_*D*_ and *S*. It shall be noted that FCS experiments are interfered by background signals (*i.e.*, *F*(*t*)_BG_), such as contaminant fluorescence, scattered light, detector dark current and after pulse. If the background signals are not correlated (*i.e.*, *G*(*τ*)_BG_ is approximately flat and equals to 1), then the shape of *G*(*τ*) and hence *τ*_*D*_ value are not affected. However, the experimentally determined *G*(0) value needs to be scaled (Hess* et al.* 2002) so that: *G*(0)_cor_ = *G*(0)<*F*(*t*)>^2^/[<F(t)> – <*F*(*t*)>_BG_]^2^, with <*F*(*t*)> and <*F*(*t*)>_BG_ being experimentally measured sample and background time-averaged photon signals respectively. If the background signals (*e.g.*, contaminant fluorescence) correlate, then *τ*_*D*_ value cannot be precisely determined. Nevertheless, if <*F*(*t*)>_BG_ is less than 5% of <*F*(*t*)>, it its acceptable to ignore background interference during an FCS experiment (Hess* et al.* 2002).

Since *S* is determined theoretically only by the confocal optical pathway (Fig. 2A), particularly the pinhole size and location, *S* value is often measured using a calibration sample (typically a 1–10 nmol/L dye solution) and then fixed during the subsequent analysis of experimental data. It is advisable to determine the *S* value for each FCS experiment session. Constant *S* values indicate stability in the optical pathway and instrument performance. Furthermore, from the experimentally determined *τ*_*D*_ value, we have:

7 $ {\tau }_{D}=\frac{{r}_{0}^{2}}{4D}\;\;. $

In Eq. 7, *D* is the diffusion coefficient of a sample molecule. τ_D_ represents the average time for sample molecules to diffuse through the FCS volume element *V* (Fig. 2B, bottom), thus it is proportional to *r*_0_ but inversely proportional to *D*. *D* is related to the hydrodynamic radius of a sample molecule or nanoparticle through the Stoke-Einstein equation:

8 $ D\left(T\right)=\frac{{K}_{\mathrm{B}}T}{6{\text{π}}\eta \left(T\right){R}_{\mathrm{H}}}\;\;. $

In Eq. 8, *K*_B_ is the Boltzmann constant, *T* is absolute temperature, *η* is solvent viscosity and *R*_H_ is the hydrodynamic radius of a sample molecule or nanoparticle. It shall be noted that both *D*(*T*) and *η*(*T*) are temperature dependent, thus results obtained from FCS experiments carried out in different buffers and at different temperatures shall be carefully calibrated. For a calibration sample of 10 nmol/L ATTO655 solution, the dye’s diffusion coefficient at 25 °C has been precisely determined (Dertinger* et al.* 2007) to be (4.26 ± 0.08) × 10^−6^ cm^2^/s. Hence this calibration sample can be used to determine *S* and *r*_0_ values of the FCS volume element *V* via Eqs. 6 and 7. Subsequently, FCS volume element *V* and molar concentration *C* of another sample can then be calculated:

9 $ V={{\text{π}}}^{\tfrac{3}{2}}{{r}_{0}}^{2}{z}_{0}\;\;,\;\;C=\frac{N}{V{N}_{\mathrm{A}}}\;\;.$

Overall, Eqs. 6–9 summarize the analytical steps for determining a fluorescent molecule’s molar concentration, its diffusion coefficient and hydrodynamic radius. This procedure typically requires a calibration sample containing a dye molecule with a known diffusion coefficient, then the molar concentrations and diffusion coefficients of other molecules can be subsequently measured. A third useful parameter, molecular brightness *λ*, can also be estimated from an FCS experiment:

10 $ \lambda =\frac{ < F\left(t\right) > }{N}\;\;. $

Molecular brightness is a function of laser power, instrument fluorescence collection efficiency, a fluorescent probe’s absorption extinction coefficient and fluorescence quantum efficiency. However, for FCS experiments carried out under the same conditions, *λ* value is a useful indicator of the biomolecular homo-oligomerization state. For example, for a protein molecule tagged with a GFP probe, its dimer shall be roughly twice as bright as its monomer.

**Figure 4:**
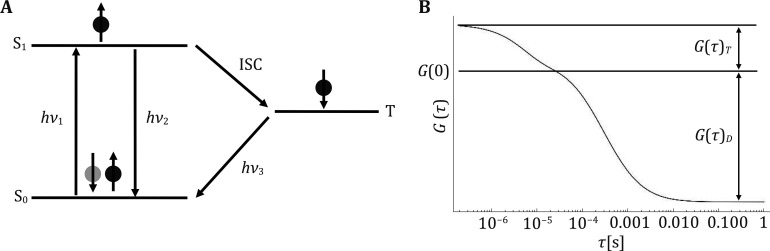
Effect of fluorophore triplet dynamics on the auto-correlation decay curve of a freely diffusing fluorescent molecule. **A** Energy diagram of single-triplet transition. **B** Effect of triplet dynamics on a fluorescence auto-correlation decay curve

As mentioned above, FCS techniques can analyze rate constants of a chemical reaction in equilibrium, provided such reaction resulting in changes in fluorescence probe’s brightness. A classic example of such a reaction, photophysical in nature, is the constant cycling of a fluorescence probe in its excited singlet or triplet states (Fig. 4A). Typically, after a fluorescent molecule absorbs the energy of an excitation photon (*hν*_1_; *h* is the Planck constant, *ν* is the frequency of the photon), an electron in the outmost orbit of the fluorescent molecule transits from its ground state (S_0_) to the first excited electronic state (S_1_). The excited electron in the S_1_ state (black circle in Fig. 4A) is unstable; typically, after a few nanoseconds (*i.e.*, fluorescence lifetime), this electron decays back to its S_0_ state, emitting a fluorescence photon (*hν*_2_) with a less than 100% probability (*i.e.*, fluorescence quantum efficiency). Very rarely, the excited electron in the S_1_ state spontaneously flips its spin direction, thus transits to the triplet state (T; *i.e.*, intersystem crossing); now the excited electron has the same spin direction as that of the pairing electron still remaining in the S_0_ state (grey circle in Fig. 4A). Quantum mechanisms thus prohibit this excited electron in the triplet state from returning to the ground state. Only after a relatively long time, typically microseconds to seconds (*i.e.*, phosphorescence lifetime), when the electron in the triplet state experienced another spontaneous spin flip, it is allowed to decay back to the S_0_ state, with a very small probability of emitting a phosphorescence photon (*hν*_3_). When the fluorophore is cycling between the S_0_ $ \leftrightarrow $ S1 states, it constantly emits fluorescence photons and appears to be bright. In contrast, when the molecule is trapped in the triplet state, it only very rarely emits a phosphorescence photon and appears to be dark. Thus, fluorophore cycling between the singlet and triplet states (*i.e.*, triplet dynamics) can be described by a simple photophysical reaction:

$ {F}^{ *}\left(\mathrm{s}\mathrm{i}\mathrm{n}\mathrm{g}\mathrm{l}\mathrm{e}\mathrm{t},\mathrm{ }\mathrm{b}\mathrm{r}\mathrm{i}\mathrm{g}\mathrm{h}\mathrm{t}\right)\leftrightarrow F\left(\mathrm{t}\mathrm{r}\mathrm{i}\mathrm{p}\mathrm{l}\mathrm{e}\mathrm{t},\mathrm{d}\mathrm{a}\mathrm{r}\mathrm{k}\right). $

The rate of such cycling is defined by a relaxation constant *τ*_*T*_ = 1/(*k*_f_ + *k*_b_), with *k*_f_ and *k*_b_ being the forward and backward reaction rate constants respectively. Incorporating the triplet dynamics, the analytical solution to the auto-correlation function for one species of fluorescent molecules diffusing in 3D-space becomes:

11 $ G\left(\tau \right)=\frac{1}{N}\cdot \frac{\left(1-T+T{e}^{-\tau /{\tau }_{T}}\right)}{1-T}\cdot \frac{1}{\left(1+\dfrac{\tau }{{\tau }_{D}}\right)\sqrt{1+\dfrac{\tau }{{\tau }_{D}{S}^{2}}}}\;\;. $

In Eq. 11, *T* indicates the fraction of *N* fluorescent molecules trapped in the triplet state. The three multiplication units on the right side of Eq. 11 can be represented by G(0), *G*(*τ*)_*T*_ and *G*(*τ*)_*D*_ respectively, thus:

12 $ G\left(\tau \right)=G\left(0\right)\cdot {G\left(\tau \right)}_{T}\cdot {G\left(\tau \right)}_{D}\;\;. $

In Eq. 12, the auto-correlation function *G*(*τ*) is broken into three parts, *G*(0), *G*(*τ*)_*T*_ and *G*(*τ*)_*D*_, with the latter two accounting for the triplet dynamics and 3D molecular diffusion respectively. Usually, *τ*_*T*_ is less than ten microseconds while *τ*_*D*_ for a biomolecule is larger than one millisecond. Under this circumstance, the *G*(*τ*)_*T*_ and *G*(*τ*)_*D*_ phases of the auto-correlation decay are well separated, and the relationship among *G*(0), *G*(*τ*)_*T*_ and *G*(*τ*)_*D*_ is shown in Fig. 4B. It shall be noted that although Eq. 11 is derived assuming a fluorophore undergoing single (bright) $ \leftrightarrow $ triplet (dark) cycling, it also applies to any chemical reaction causing the fluorophore to become brighter or dimmer. Since FCS experiments require fluorescent labeling and essentially all fluorophores undergo triplet dynamics, Eq. 11 is a universal fitting model for analyzing FCS data of single species molecular diffusing in 3D space. Of course, we can ignore *G*(*τ*) data under a certain *τ* threshold (*i.e.*, *τ* < 10 μs); in this case, Eq. 6 is sufficient to analyze single species molecular diffusion in 3D space with minimum interference from a fluorophore’s triplet dynamics.

If there are *i*^th^ species of fluorescent molecules freely diffusing in a sample solution and these molecules do not interact with each other, then the analytical solution to the auto-correlation function is:

13 \begin{equation*}\begin{split} 
G\left(\tau \right)\;&=\frac{{\displaystyle\sum }_{i=1}{{\lambda }_{i}}^{2}{N}_{i}\cdot {{G}_{i}\left(\tau \right)}_{D}}{\left({\displaystyle\sum }_{i=1}{\lambda }_{i}{N}_{i}\right)^{2}}\\&=
{\displaystyle\sum }_{i=1}{f}_{i}\cdot {{G}_{i}\left(\tau \right)}_{D}\;\&\;{f}_{i}=\frac{{{\lambda }_{i}}^{2}{N}_{i}}{\left({\displaystyle\sum }_{i=1}{\lambda }_{i}{N}_{i}\right)^{2}}\;\;.
\end{split}\end{equation*}

In Eq. 13, *λ*_*i*_ is the brightness, *N*_*i*_ is the average number within the FCS volume element, and *G*_*i*_(*τ*)_*D*_ is the diffusional auto-correlation decay of the *i*^th^ fluorescent molecule species, respectively. *f*_*i*_ represents the contribution of the *i*^th^ fluorescent species to the overall fluorescence auto-correlation decay curve. Importantly, such contribution is dependent on the square of molecule brightness (*λ*_*i*_^2^). Thus, bright molecules contribute disproportionally to the overall auto-correlation decay curve for a sample containing multiple fluorescent species. For a simple biomolecular binding reaction, such as antigen-antibody binding, Eq. 13 becomes:

14 \begin{equation*}\begin{split} 
G\left(\tau \right)=\;&\dfrac{{\mathrm{\lambda }}_{1}^{2}{N}_{1}}{({\mathrm{\lambda }}_{1}{N}_{1}+{\mathrm{\lambda }}_{1}{N}_{2}{)}^{2}}\cdot \dfrac{1}{\left(1+\dfrac{\tau }{{\tau }_{\text{1}}}\right)\sqrt{1+\dfrac{\tau }{{\tau }_{\text{1}}{S}^{2}}}}\\& +\dfrac{{\mathrm{\lambda }}_{2}^{2}{N}_{2}}{({\mathrm{\lambda }}_{1}{N}_{1}+{\mathrm{\lambda }}_{2}{N}_{2}{)}^{2}}\cdot \dfrac{1}{\left(1+\dfrac{\tau }{{\tau }_{\text{2}}}\right)\sqrt{1+\dfrac{\tau }{{\tau }_{\text{2}}{S}^{2}}}}\;\;. 
\end{split}\end{equation*}

In Eq. 14, Molecule One could be the smaller and fluorescently labeled antigen (Ag*; the antibody is unlabeled), and Molecule Two the antigen–antibody binding product (Ag*Ab); *τ*_1_ and *τ*_2_ are then characteristic diffusion correlation times for Ag* and Ag*Ab respectively. In this case, *τ*_2_ shall be at least ~60% larger than *τ*_1_ for the FCS analysis to distinguish two different diffusional species (*i.e.*, Ag* and Ag*Ab) in the sample solution (Meseth* et al.* 1999). Diffusion correlation time *τ*_*D*_ scales linearly with molecular hydrodynamic radius R_H_ (Eqs. 7 and 8), and *R*_H_ can be roughly estimated to be the cubic root of molecule weight (MW). Thus, MW of Ag*Ab needs to be at least ~4 folds bigger than that of Ag*. If this condition is satisfied, FCS experiments have been successfully implemented to determine the dissociation constants (*K*_D_) of biomolecular binding reactions (Tetin* et al.* 2013; Luo* et al.* 2021).

**Figure 5:**
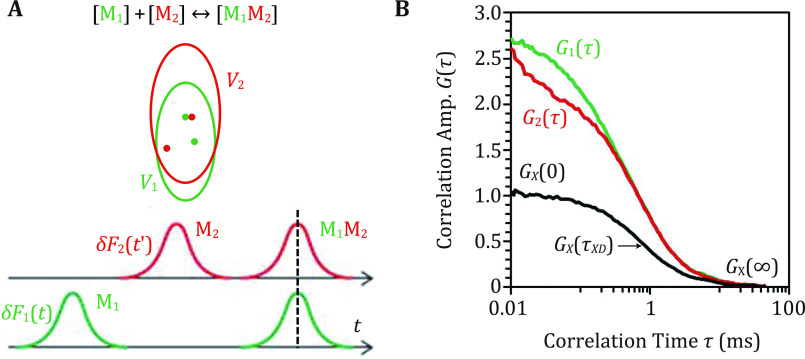
Principle of due-color fluorescence cross-correlation spectroscopy (dcFCCS). **A** Ideal single molecule signals obtained from two partially overlapping dcFCCS volume elements. **B** Representative two fluorescence auto-correlation decay curves and one fluorescence cross-correlation decay curve obtained from a single dcFCCS experiment

If the two interacting molecules (*e.g.*, M_1_ and M_2_) have similar sizes, then we need the fluorescence cross-correlation spectroscopy (FCCS) technique to quantitatively evaluate their binding reaction (Fig. 5). These two molecules are typically labeled with two fluorophores with ideally widely separated fluorescence emission spectra: *e.g.*, M_1_ labeled with the green Alexa488 and M_2_ labeled with the red ATTO655 fluorophores. In this case, fluorescence cross-correlation analysis is specifically named dual-color FCCS (dcFCCS). dcFCCS experiment can be carried out using the same confocal optical configuration depicted in Fig. 2A, using typically two lasers to excite the two fluorophores and two detection channels for the green *F*_1_(*t*) and red *F*_2_(*t*) fluorescence signals respectively. Similarly, there are two FCS volume elements (*i.e.*, *V*_1_ and *V*_2_), and *V*_2_ > *V*_1_ because red light is focused less tightly by the optical pathway compared to the green light (Fig. 5A). Furthermore, because of the inevitable chromatic aberrations in both fluorescence excitation and detection, *V*_1_ and *V*_2_ do not completely overlap along the optical axis (Fig. 5A). Nevertheless, this dcFCCS setup offers opportunities to qualitatively and quantitatively study biomolecular interactions *in vivo* and *in vitro*.

We can intuitively understand the principles of dcFCCS analysis using ideal SMF signals similar to that discussed in Fig. 2 and Fig. 3. Because the temporal resolution of typical SPAD detectors used in dcFCCS experiment is tens of nanoseconds, while the typical dwelling time for a biomolecule in the FCS volume element is larger than one millisecond. Therefore, the detectors are able to temporally discriminate SMF signals *δF*_1_(*t*) and *δF*_2_(*t*) originally from M_1_ and M_2_ respectively. If M_1_ and M_2_ do not bind together and thus diffuse independently (separated green and red dots), their corresponding *δF*_1_(*t*) and *δF*_2_(*t*') will be detected at different times (Fig. 5A). In contrast, M_1_ and M_2_ diffuse together within the binding product M_1_M_2_; thus *δF*_1_(*t*) and *δF*_2_(*t*') will be detected approximately at the same time. The former case results in little cross-correlation and the latter significant cross-correlation, which can be quantitatively analyzed using the cross-correlation function:

15 $ {\mathrm{G}}_{x}\left(\tau \right)=\frac{ < {F}_{1}\left(t\right){F}_{2}\left(t+\tau \right) > }{ < {F}_{1}\left(t\right){ >  < F}_{2}\left(t\right) > }=\frac{ < \delta {F}_{1}\left(t\right)\delta {F}_{2}\left(t+\tau \right) > }{ < {F}_{1}\left(t\right){ >  < F}_{2}\left(t\right) > }+1\;\;. $

There shall be no cross-correlation between the photophysics (*i.e.*, singlet-triplet cycling; Fig. 4) of the different fluorophores used to label M_1_ and M_2_. Thus, we only need to find the analytical solution to the cross-correlation function (Eq. 15) in terms of M_1_ and M_2_ diffusion in 3D space. As the first step, we assume the ideal case: *i.e.*, *V*_1_ = *V*_2_ = *V* and both FCS volume elements overlaps perfectly. In this case (Schwille* et al.* 1997; Wohland* et al.* 2020),

16 $ {G}_{X}\left(\tau \right)={G}_{X}\left(0\right)\cdot \frac{1}{\left(1+\dfrac{\tau }{{\tau }_{\text{XD}}}\right)\sqrt{1+\dfrac{\tau }{{\tau }_{\text{XD}}{S}^{2}}}}\;\;. $

Equation 16 is very similar to Eq. 6, except *G*_*X*_(0) and *τ*_*XD*_ are related respectively to the molar concentration and hydrodynamic radius of the binding product M_1_M_2_ in a dcFCCS experiment.

17 \begin{equation*}\begin{split} 
{G}_{X}\left(0\right)\;&=\frac{{N}_{X}}{\left({N}_{1}+{N}_{X}\right)\left({N}_{2}+{N}_{X}\right)}\\&=
\frac{\left[{M}_{1}{M}_{2}\right]}{V{N}_{A}(\left[{M}_{1}\right]+\left[{M}_{1}{M}_{2}\right])(\left[{M}_{2}\right]+\left[{M}_{1}{M}_{2}\right])}\;\;.
\end{split}\end{equation*}

Here, *N*_1_, *N*_2_ and *N*_*X*_ are the average numbers of M_1_, M_2_ and M_1_M_2_ molecules in the volume element *V* respectively, from which molar concentrations of [M_1_], [M_2_] and [M_1_M_2_] can be calculated; *N*_A_ is the Avogadro constant. A typical dcFCCS experiment also produces two auto-correlation decay curves *G*_1_(0) and *G*_2_(0) (Fig. 5B), resulting from auto-correlation analysis of the fluctuating fluorescence signals *F*_1_(*t*) and *F*_2_(*t*) respectively. Using Eq. 11 to quantitatively analyze *G*_1_(*τ*) and *G*_2_(*τ*), which produces the experimentally determined *G*_1_(0) and *G*_2_(0) values:

18 $\left\{\begin{aligned} &
{G}_{1}\left(0\right)=\frac{1}{{N}_{1}+{N}_{X}}=\frac{1}{V{N}_{\mathrm{A}}\left(\left[{M}_{1}\right]+\left[{M}_{1}{M}_{2}\right]\right)}\\&
{G}_{2}\left(0\right)=\frac{1}{{N}_{2}+{N}_{X}}=\frac{1}{V{N}_{\mathrm{A}}(\left[{M}_{2}\right]+\left[{M}_{1}{M}_{2}\right])}
\end{aligned} \right.\;\;.$

As expected *G*_1_(0) and *G*_2_(0) are inversely proportional to the total numbers of green (*N*_1_ + *N*_*X*_) and red (*N*_2_ + *N*_*X*_) molecules respectively, regardless of whether these molecules are in the free (*N*_1_, *N*_2_) or associated form (*N*_*X*_). Since both (*N*_1_ + *N*_*X*_) and (*N*_2_ + *N*_*X*_) remain constant for a biomolecular binding reaction, *G*_*X*_(0) values acquired during a dcFCCS experiment reflect the real-time formation of the molecular complex M_1_M_2_ and can be used to monitor the reaction kinetics. dcFCCS experiment is also very useful for determining dissociation constant (*K*_D_) for the biomolecular binding reaction, particularly in live cells. From Eqs. 17 and 18, it can be shown:

19 $ {K}_{\mathrm{D}}=\frac{\left[{M}_{1}\right]\left[{M}_{2}\right]}{{[M}_{1}{M}_{2}]}=\frac{\left[{\mathrm{G}}_{1}\left(0\right)-{\mathrm{G}}_{X}\left(0\right)\right]\left[{\mathrm{G}}_{2}\left(0\right)-{\mathrm{G}}_{X}\left(0\right)\right]}{V{{N}_{\mathrm{A}}G}_{1}\left(0\right){G}_{2}\left(0\right){G}_{X}\left(0\right)}\;\;. $

Finally, the diffusion coefficient *D*_*X*_ and hydrodynamic radius *R*_*X*H_ of the molecular complex M_1_M_2_ can be estimated using the experimentally determined value of *τ*_*XD*_:

20 $ {\tau }_{XD}=\frac{{r}_{0}^{2}}{{4D}_{X}}\;\;, $

21 $ {D}_{X}\left(T\right)=\frac{{K}_{\mathrm{B}}T}{6{\text{π}}\eta \left(T\right){R}_{X\mathrm{H}}}\;\;.$

Equations 16–21, derived from the assumption of equal and ideal overlapping of volume elements *V*_1_ and *V*_2_, can be used to qualitatively evaluate dcFCCS data obtained from actual experiments. This approach is still very useful for biological studies, particularly in live cells, when relative but not absolute changes in molecular properties (*e.g.*, concentration, interaction) are often sufficient for hypothesis testing. Nevertheless, quantitative evaluation of dcFCCS data requires careful elimination of interference factors such as non-ideal overlapping of the green and red volume elements, spectral cross-talk between the fluorescence signals, non-correlated background, non-fluorescent binding partners and free fluorophores. Detailed discussion about experimental designs and analytical treatments to minimize those interferences are beyond the scope of this review. Interested readers can find excellent literature sources discussing these issues in exquisite detail (Weidemann and Schwille 2013; Wohland* et al.* 2020).

In summary, FCS techniques analyze the temporal relationship between a fluorescence fluctuation occurring at time *t* [*i.e.*, *δF*(*t*)] and another occurring at a short time interval *τ* later [*i.e.*, *δF*(*t* + *τ*)], with FACS and dcFCCS methods working with fluorescence signals of a single color and two separated colors respectively. Such analysis yields quantitative information about molecular concentration and diffusion coefficient, from which hydrodynamic radius and binding affinity (*K*_D_) can be further derived. Nevertheless, FCS techniques are insufficient in resolving a heterogeneous sample solution containing molecular species of different fluorescence brightness (Eq. 13). Thus, a complementary set of analytical methods, utilizing the same raw fluorescence photon data but different temporal information, have been developed to quantitatively analyze molecular species of different brightness (Macdonald* et al.* 2013; Wu* et al.* 2013). Based on the same statistical principle but using different mathematical approaches, photon counting histogram (PCH; Chen* et al.* 1999; Perroud* et al.* 2005 and fluorescence intensity distribution analysis (FIDA; Kask* et al.* 1999; Palo* et al.* 2000) were independently developed. PCH and FIDA analyze the probability distribution (histogram) of numbers of photons detected within a short time bin *T*. *T* is typically much smaller than *τ*_*D*_, so that fluorescent molecules under investigation can be regarded as stationary within the FCS volume element (Fig. 2B, bottom) for such analysis. The intuitive interpretation of the PCH and FIDA methods is that the brighter molecular species will yield more photons within a fixed time bin *T*. Considering molecular diffusion, the time-integrated fluorescence cumulant analysis (TIFCA) method was further developed (Muller 2004; Wu and Muller 2005), which allows arbitrarily larger time bin *T* producing much higher signal-to-noise data for photon histogram analysis. Together, PCH, FIDA and TIFCA methods have been used to resolve molecular species of different fluorescence brightness and their respective concentrations in heterogeneous solutions. These techniques are especially useful in quantitatively determining the homo- and hetero-oligomerization states of molecules tagged with fluorescence proteins in live cells (Kask *et al*. 1999; Palo *et al*. 2000).

**Figure 6:**
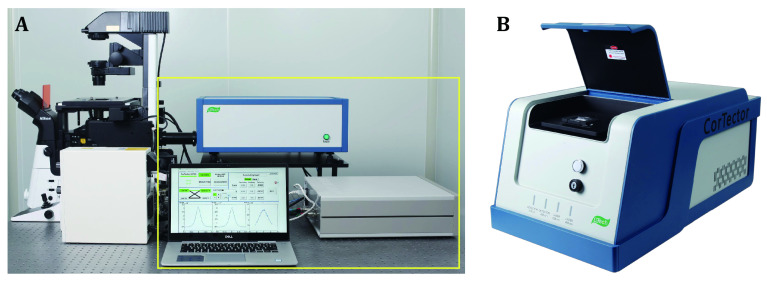
Representative commercial FCS instrument. **A** FCS module (within the yellow box) attached to an inverted fluorescence microscope. **B** A benchtop FCS unit capable of fluorescence auto- and cross-correlation analysis

Finally, Many FCS instruments used in basic research are home-built, which suffer primarily from difficulties related to the alignment of the optical pathway, particularly precise positioning of the confocal pinhole that is critically important for each FCS experiment (Hess and Webb 2002). Additional inconveniences include data analysis, which requires users to understand the principles of FCS techniques, as well as fluorophore photophysics (*e.g.*, triplet dynamics, photobleaching, fluorescence saturation) that also interfere with correlation data. Thus, commercial FCS instruments equipped with automatic confocal pinhole alignment and semi-automatic data analysis software are urgently needed. Figure 6 shows two types of FCS instruments: microscope-based module (Fig. 6A) and self-contained benchtop unit (Fig. 6B). Both instruments are equipped with automatic confocal pinhole alignment, and the manufacturer (LightEdge Technologies Ltd.; Zhongshan, Guangdong, China) also provides custom software development for specific data analysis needs. The microscope-based FCS module is suitable for live-cell studies, but is more expensive to purchase and maintain, as well as requiring a microscope dedicated room and professionally trained users. The benchtop FCS unit is much easier to use and maintain, but is currently restricted to solution or cell lysate samples. Benchtop FCS instrument with both auto-correlation and cross-correlation analytical capabilities is only available at LightEdge Technologies. The microscope-based FCS systems are also sold by Zeiss (Oberkochen, Germany), PicoQuant (Berlin, Germany) and ISS (Champaign, Illinois, USA). The FCS capabilities are optional to some late models of the Zeiss confocal microscopes, which are convenient for live-cell studies. The PicoQuant FCS capabilities are based on time-correlated single-photon counting (TCSPC), which requires expansive picosecond (ps) pulsed lasers and ps-resolution TCSPC data acquisition card. Yet, TCSPC offers additional FCS capabilities such as anti-bunching and fluorescence lifetime correlation spectroscopy (FLCS). The FCS system offered by ISS comes with additional scanning FCS and PCH capabilities. All the microscope-based FCS systems offered by foreign vendors are quite expansive, with the entire package typically costs above three million RMB. Interested readers can find out more about those FCS systems on the manufacturers’ websites.

## FCS TECHNIQUES APPLIED TO LLPS RESEARCH *IN VIVO* AND *IN VITRO*

As adequately shown above, both the fluorescence auto-correlation (FACS or FCS) and cross-correlation (FCCS) techniques are capable of providing key evidences (*i.e.*, local concentration, homo- or hetero-interaction, dissociation constant, diffusion coefficient, hydrodynamic radius, *etc*.) for revealing molecular mechanisms of specific cellular functions, particularly in live cells. Unsurprisingly, these capabilities found widespread applications in studies of biomolecular phase separation, first in the lipid bilayer (Chiantia* et al.* 2009; Eggeling* et al.* 2009; He and Marguet 2011; Korlach* et al.* 1999; Mueller* et al.* 2013) and more recently in cell cytosol/nucleus. A whole new class of membraneless cellular organelles, also named condensates or droplets, have been discovered. Such organelles are hypothesized to be the results of biomolecular LLPS. Important features of these cellular condensates are their apparent abilities to concentrate specific proteins and nucleic acids in liquid droplets, within which biomolecular diffusion is restricted to various degrees. These novel organelles play important roles in various cellular functions (Alberti* et al.* 2019; Shin and Brangwynne 2017), yet their molecular mechanisms still need to be further explored.

**Figure 7:**
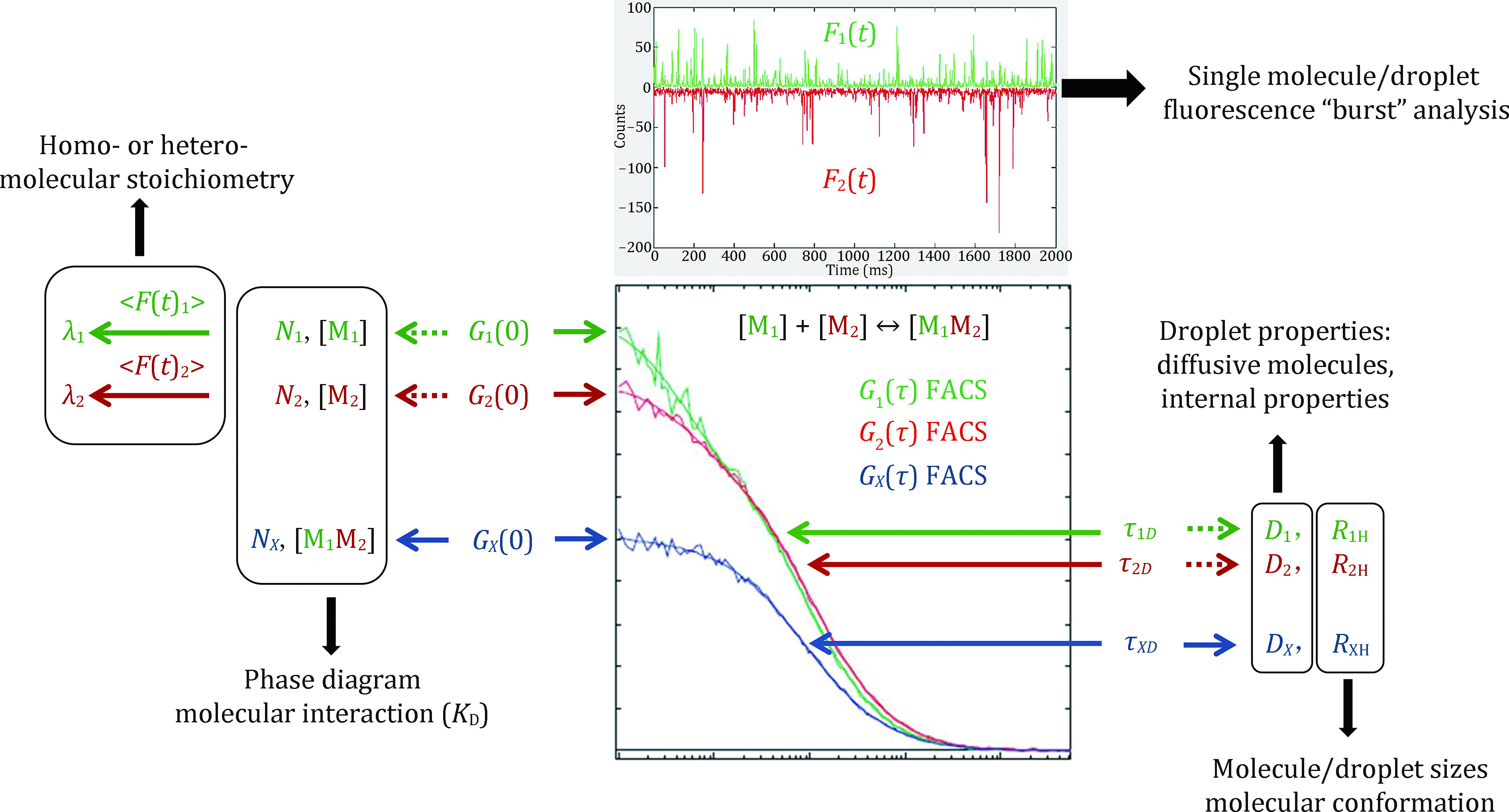
FCS techniques applied to biomolecular liquid–liquid phase separation research

Multivalent weak protein–protein and/or protein–nucleic acid interactions are proposed to be an important driving force for LLPS. In Fig. 7, we use a hypothetic dcFCCS experiment on a biomolecular binding reaction to demonstrate the multiple capabilities of FACS and FCCS for investigating molecular properties important for LLPS. A typical dcFCCS experiment produces two fluctuating fluorescence intensities *F*_1_(*t*) and *F*_2_(*t*), from which two auto-correlation *G*_1_(*τ*) and *G*_2_(*τ*) plus one cross-correlation *G*_*X*_(*τ*) decay curves can be derived. The auto-correlation and cross-correlation curves can be quantitatively analyzed using Eq. 11 and 16 respectively. Thus, from the *G*_1_(0), *G*_2_(0) and *G*_*X*_(0) amplitudes, average numbers *N*_1_, *N*_2_ and *N*_*X*_ of the three molecular species within the FCS volume element *V* and their respective molar concentrations [M_1_], [M_2_] and [M_1_M_2_] can be deducted (Eqs. 17 and 18). The molar concentrations can be used to construct a phase diagram of LLPS and determine *K*_D_ value (Eq. 19) of the biomolecular interaction, particularly in live cells. From the average fluorescence intensities <*F*_1_(*t*)> and <*F*_2_(*t*)> and corresponding *N*_1_ and *N*_2_, molecular brightness *λ*_1_ and *λ*_2_ are calculated (Eq. 10), which can be used to estimate homo- or hetero-molecular stoichiometry with a molecular cluster or nanoscale droplet. *G*_1_(*τ*), *G*_2_(*τ*) and *G*_*X*_(*τ*) decay rates are characterized by *τ*_1*D*_, *τ*_2*D*_ and *τ*_*XD*_ respectively, from the latter values we can derive molecular or nanoscale droplet diffusion coefficients *D*_1_, *D*_2_ and *D*_*X*_, as well as the corresponding hydrodynamic radii *R*_1H_, *R*_2H_ and *R*_*X*H_ (Eqs. 7, 8, 20 and 21). It is essential to establish the diffusive nature of constitutive molecules within droplets, thus proving the existence of the liquid phase within these condensates. Quantitative *D*-values measured for molecules of different sizes or chemical properties can be used to further explore the internal properties (*e.g.*, pore size, multivalent interaction) of the droplets. Due to the limited size of the FCS volume element *V* (*r*_0_ < 500 nm), hydrodynamic radii of only molecular clusters or nanoscale droplets can be measured. For micron-sized droplets, FCS techniques are used to explore molecular properties within, outside and on the boundary of these condensates. Hydrodynamic radii are also indicative of molecular conformations, particularly when FCS techniques are used in combination with the fluorescence resonance energy transfer (FRET) methods *(i.e.*, FCS-FRET) (Felekyan* et al.* 2013; Wohland* et al.* 2020). Finally, using very diluted molecule or droplet samples, the raw photon streaming data *F*_1_(*t*) and *F*_2_(*t*) containing single-molecule or single droplet signals (*i.e.*, “burst” signals). Sophisticated algorithms have been developed to analyze such “burst” signals (*i.e.*, “burst” analysis), which allows single-molecule or single-droplet studies in a solution sample or a single live cell (Lerner* et al.* 2018, 2021). These algorithms are partially implemented in the data analysis software (*i.e.*, Correlation Analysis) developed for the FCS instruments shown in Fig. 6. Therefore, a complete set of hardware and software tools, as described in this review article, are now commercially available to researchers in the LLPS field. Below, we review FCS applications to LLPS research in live cells and in aqueous solutions (Table 1).

**Table 1:** Applications of FCS techniques to research of biomolecular liquid–liquid phase separation *in vivo* and i*n intro*

	Research subjects	Molecular properties	FCS techniques
Live-cell applications	Centrosome (Mahen* et al.* 2011)	Diffusion coefficient	FCS, RICS, line-scanning FCS
	DNA transcription site (Chong* et al.* 2018)	Absolute concentration^a^	FCS
	Tight junction (Beutel* et al.* 2019)	Diffusion coefficient, absolute concentration, molecular brightness	FCS, scanning FCS
	Inner centromere(Trivedi* et al.* 2019)	Diffusion coefficient	FCS
	Nucleolus (Zhu* et al.* 2019)	Diffusion coefficient, absolute concentration	FCS
	Pre-autophagosome structure (Fujioka* et al.* 2020)	Diffusion coefficient	FCS
	Stress granule, P-body (Sanders* et al.* 2020)	Absolute concentration	FCS
	Lipid droplet-lipid droplet contact site (Lyu* et al.* 2021)	Diffusion coefficient	FCS
	Photoinducible “corelets” (Bracha* et al.* 2018; Shimobayashi* et al.* 2021)	Diffusion coefficient, absolute concentration	FCS
	Prion-like RNA-binding proteins (Kaur* et al.* 2021)	Diffusion coefficient	FCS
	Ribosome crowding induced LLPS (Delarue* et al.* 2018)	Diffusion coefficient	FCS
	Heterochromatin (Erdel* et al.* 2020)	Translational diffusion, rotational diffusion, molecular concentration, droplet size and abundance	FCS, Pol-FCS, ICS
*In vitro* applications	Polypeptide-DNA (Shakya and King 2018a)	Diffusion coefficient, molecular interaction	FCS, FCCS
	Histone proteins-DNA (Shakya and King 2018b)	Diffusion coefficient	STED-FLCS^b^
	Histone proteins-nucleosome (Shakya* et al.* 2020)	Absolute concentration, diffusion coefficient	FCS
	Nucleoprotien-RNA (Alshareedah* et al.* 2020)	Diffusion coefficient	FCS
	Polypeptide-ssDNA (Alshareedah* et al.* 2021)	Diffusion coefficient	FCS
	Prion-like domains (Martin* et al.* 2020, 2021)	Absolute concentration, molecular brightness, diffusion coefficient	FCS, Dual-focus FCS
	Poly(Lys)-Poly(rU),Poly(Arg)-Poly(rU) (Fisher and Elbaum-Garfinkle 2020)	Absolute concentration, molecular interaction (dissociation constant *K*_D_)	FCS, Dual-focus FCS
	Peptide-Poly(rU) (Kaur* et al.* 2021)	Molecular interaction	FCS
	*In vitro* model of stress granule formation (Guillen-Boixet* et al.* 2020)	Diffusion coefficient (hydrodynamic radius)	FCS, scanning FCS
	ySmF-peptides (Peng* et al.* 2020)	Molecular diffusion (hydrodynamic radius), molecular brightness (binding stoichiometry), molecular interaction (dissociation constant *K*_D_)	dcFCCS
	*C. elegans* protein LAF-1 (Wei* et al.* 2017)	Molecular concentration, molecular diffusion (diffusion coefficients, viscosity, second viral coefficients, mesh size)	Ultrafast-scanning FCS
	Microtubule-associated Tau protein (Wen* et al.* 2021)	Molecular diffusion, molecular brightness	FRET-FCS
	SARS-CoV-2 N-protein (Cubuk* et al.* 2021)	Molecular dynamics	Nanosecond FRET-FCS
^a^ Absolution concentration refers to molar concentration calculated from the average number of fluorescent molecules detected from the FCS volume element (Eq. 9). Such a concentration is determined without constructing a reference concentration curve that is typically required using the traditional method of the spectrophotometer. Thus, the FCS-determined molar concentration is also called absolute concentration.			
^b^ FLCS: fluorescence lifetime correlation spectroscopy, an interdisciplinary technique combining fluorescence lifetime fluorescence spectroscopy and fluorescence correlation spectroscopy (Ghosh* et al.* 2018; Machan* et al.* 2014).			

FCS can measure absolute concentrations of fluorescently labeled molecules and their diffusion coefficients within or outside of biomolecular condensates, thus it is an essential technique, together with fluorescence recovery after photobleaching (FRAP), for establishing the liquid phase interior of the membraneless organelles resulting from LLPS, particularly in live cells (Table 1) (Beutel* et al.* 2019; Chong* et al.* 2018; Fujioka* et al.* 2020; Lyu* et al.* 2021; Mahen* et al.* 2011; Sanders* et al.* 2020; Trivedi* et al.* 2019; Zhu* et al.* 2019). Using photo-inducible oligomers (*i.e.*, “corelets”) to regulate LLPS at specific sites in live cells, Bracha *et al.* were able to construct *in vivo* phase separation diagrams based on FCS’s capability of quantifying biomolecular concentrations in single live cells (Bracha* et al.* 2018). Using FCS to measure diffusion coefficients of “corelets” and liquid droplets, the authors proposed a “diffusive capture mechanism”: *i.e.* slower diffusing oligomers (seeds) recruit and concentrate interacting biomolecules so that local LLPS occurs even when global biomolecular concentrations are below saturation level (Bracha* et al.* 2018). Using the same “corelets” and FCS tool, this research group further demonstrated that LLPS in live cells can be described by the “classical nucleation theory”, but the efficacy of nucleation is tuned by biomolecular features (*e.g.*, specific amino acid sequences in intrinsically disorder regions) (Shimobayashi* et al.* 2021). Finally, FCS also assisted in the investigations of LLPS of prion-like RNA-binding proteins and LLPS induced by intracellular ribosome crowding through measurements of molecular diffusion in single live cells.

*In vitro* FCS studies using solution samples provided substantial mechanistic insights about LLPS. Using FCS technique to measure ssDNA diffusion in and out of liquid droplets, Shakya and King investigated LLPS induced by multivalent interactions between single- or double-strand DNA and cationic polypeptides. The authors found that flexible DNA sequence, but not DNA charge distribution, is the dominant factor facilitating LLPS (Shakya and King 2018a). The authors further showed that a mixture of the linker and structural histone proteins spontaneously form liquid droplets in presence of dsDNA (Shakya and King 2018b). Using a stimulated emission depletion (STED) super-resolution microscope to precisely reduce the FCS volume element (*i.e.*, diameter 2*r*_0_ = 35–224 nm), they measured diffusion coefficients of various fluorophores and fluorescently labeled ssDNA in the histone–DNA droplets at the STED controlled spatial scales (Shakya and King 2018b). These results indicate that molecular diffusion in the histone-DNA droplets is not Fickian, instead of the non-Fickian diffusion (*i.e.*, diffusion rates vary at different nanometer spatial scales) results from cation−π interactions between biomolecules participating in the LLPS (Shakya and King 2018b). Later, the same research group demonstrated that the linker histone protein H1 plays a dominant role in promoting LLPS in presence of intact nucleosomes of various lengths, and used molecular concentrations measured by FCS to construct phase separation diagrams (Shakya* et al.* 2020). H1 protein interacts with heterochromatin protein HP1a, suggesting a mechanism of transcription-silenced heterochromatin formation through LLPS (Shakya* et al.* 2020). Alshareedah *et al.* investigated LLPS induced by mixtures of an arginine-rich nucleoprotein (protamine) with an excessive amount of single-strand RNA (Alshareedah* et al.* 2020). Remarkably, they observed micron-sized hollow vesicles with their lipid-free rims formed through protein–RNA LLPS, which was established through FCS measurements of molecular diffusion within and outside of the rims (Alshareedah* et al.* 2020). Later, this research group investigated liquid droplets formed through multivalent interactions between the cationic polypeptide and ssDNA (Alshareedah* et al.* 2021). FCS experiments revealed a consistent mesoscopic core structure of the droplets formed with different peptide–ssDNA compositions, as evidenced by invariant diffusion coefficients of the component peptide and ssDNA in these biomolecular condensates (Alshareedah* et al.* 2021). The multi-functional nature of the FCS technique was well demonstrated in the research about LLPS of prion-like domains; FCS measures molecular diffusion within and outside of liquid droplets, degrees of molecular brightness are used to infer oligomerization state, and molecular concentrations are used to construct phase separation diagrams (Martin* et al.* 2020, 2021). Studying an *in vitro* model of stress granule formation, FCS was used to measure the diffusion coefficient of a key constitute protein within liquid droplets, from which its molecular hydrodynamic radius was calculated (Guillen-Boixet* et al.* 2020). Finally, in multiple research projects, FCS techniques have been used to determine molecular interactions related to LLPS, either qualitatively (Kaur* et al.* 2021; Shakya and King 2018a) or quantitatively (Fisher and Elbaum-Garfinkle 2020).

As demonstrated above, FCS and related fluorescence correlation techniques are able to quantitatively determine multiple molecular properties important for LLPS *in vitro* or *in vivo*, including molecular concentration, molecular brightness (oligomerization state), molecular diffusion (hydrodynamic radius), and molecular interaction (*K*_D_). With skillful implementation, other advanced FCS techniques can be especially useful tools for unraveling molecular mechanisms of LLPS, providing mechanistic insights that are inaccessible to other research methods. Using dcFCCS technique, Peng *et al.* characterized the formation of nanoscale clusters or condensates beyond the resolving power of conventional fluorescence microscopy (Peng* et al.* 2020). The authors engineered a multivalence-driven phase separation system using the *Saccharomyces cerevisiae* SmF (ySmF) protein fused to either one of the two interacting peptides labeled with green (Alexa488) and red (Cy5) fluorophores respectively. dcFCCS can then measure the sizes and growth rates of the ySmF-peptide driven formation of LLPS droplets. Liquid droplets as small as ~23 nm formed with 50 nmol/L ySMF-peptide concentration were characterized, which is beyond the detection limits of other research tools such as turbidity measurement, dynamic light scattering, *etc*. (Peng* et al.* 2020). Using FCS-measured molecular brightness, binding stoichiometry between the two interacting peptides can be determined through analysis of raw photon streaming data obtained from the same dcFCCS experiments (Peng* et al.* 2020). Combined FACS-dcFCCS experiments, binding affinity (*K*_D_) can also be derived for the interacting peptides within the nanoscale droplets (Peng* et al.* 2020). Precise FCS measurements are sensitive to refractive index mismatch between the immersion medium (*e.g.*, oil) of the microscope objective and that of the liquid droplet. Thus, Wei *et al.* developed an ultrafast scanning FCS (usFCS) technique that was used to precisely determine the FCS volume element *V*, thus accurately measuring the *V*-dependent results such as diffusion coefficient and molecular concentration (Eqs. 7–9) (Wei* et al.* 2017). Using this improved tool to investigate the disordered protein LAF-1 participating in P-granule formation, the authors established phase separation diagrams (*i.e.*, binodals), and measured molecular concentrations, diffusion coefficients, second viral coefficients, viscosity and mesh sizes within the biomolecular condensates (Wei* et al.* 2017). They found highly permeable, low density (semi-dilute) liquid droplets with internal mesh sizes of ~3–8 nm, which is a key feature driven by the conformation flexibility of the participating intrinsically disordered proteins (IDPs). The importance of protein conformation in LLPS is further examined through a combined single-molecule fluorescence resonance energy transfer (smFRET) and FRET-FCS approach (Wen* et al.* 2021). The authors investigated LLPS of the microtubule-associated protein Tau in the presence of the crowding reagent polyethylene glycol (PEG). To increase the detection sensitivity to nanoscale Tau clusters, the FRET-FCS technique was used: (1) two populations of Tau proteins were respectively labeled with donor fluorophore Alexa488 and acceptor fluorophore Alexa647; (2) these labeled Tau samples were then mixed and incubated together at final Tau concentrations of 0.1–500 nmol/L; (3) FCS experiments were carried out using fluorescence excitation at the Alexa488 channel and fluorescence detection at the Alexa647 channel (Wen* et al.* 2021). This way only nanoscale clusters formed through intermolecular interaction between Alexa488- and Alexa647-labeled Tau proteins were detected using the FRET-FCS technique. Using a similar FRET-FCS technique, it has been shown that as small as 0.5% FRET-capable clusters can be quantified among a sea of fluorescently labeled non-FRET monomers (Wennmalm* et al.* 2015). It was found that nanoscale Tau clusters, which could serve as nucleating core for subsequent droplet formation, exist even at sub-nanomolar protein concentration; smFRET studies then revealed that Tau proteins in the liquid droplets exhibit an extended conformation that is essential for LLPS (Wen* et al.* 2021). Besides FRET-FCS, the authors also carried out separated time-resolved fluorescence anisotropy studies of fluorescently labeled Tau proteins in the droplets, with results indicating that rotational mobility of Tau protein is also reduced through intermolecular interactions within the liquid droplets (Wen* et al.* 2021). In fact, within the LLPS condensates, not only the translational mobility (milliseconds) but also the rotational mobility (nanoseconds) can be simultaneously investigated using the polarization-FCS technique (Pol-FCS) (Erdel* et al.* 2020). Erdel *et al.* investigated the potential LLPS nature of heterochromatin foci in live cells, and the role of heterochromatin protein 1 (HP1) in foci formation (Erdel* et al.* 2020). Compared to translational mobility accessible to the conventional FCS method, rotational mobility is a more sensitive measurement of biomolecular homo- or hetero-oligomerization states, as well as local viscosities *in vitro* or *in vivo*. *In vitro* studies revealed that HP1 can form liquid droplets in the presence of DNA. However, the rotational mobility of HP1 is unchanged in heterochromatin foci or in the nucleoplasm of live cells. Furthermore, the size, global accessibility, and compaction of heterochromatin foci are independent of HP1, and heterochromatin foci lack a separated pool of liquid HP1 molecules in live cells. These results indicate that heterochromatin foci resemble collapsed polymer globules rather than the classic LLPS droplets (Erdel* et al.* 2020). In this paper (Erdel* et al.* 2020), the authors further used the image correlation spectroscopy (ICS) technique to quantitatively evaluate heterochromatin foci numbers and sizes in live cells. While FCS techniques reveal hidden patterns of fluorescence signal fluctuations (reflecting underlying molecular mechanisms) in the temporal domain, ICS techniques discover similar patterns in the spatial domain (Hebert* et al.* 2005; Kolin and Wiseman 2007; Petersen* et al.* 1993). The precision of ICS derived parameters is dependent on the resolution of the specific microscopy imaging method used, thus this study acquired digital images through a super-resolution STED microscope (Erdel* et al.* 2020).

Intrinsically disordered proteins (IDPs) or regions (IDRs) are key players of LLPS (Shin and Brangwynne 2017; Uversky 2017), as abundantly demonstrated in the literature discussed above. By definition, IDPs/IDRs assume a wide range of structural conformations undergoing rapid interconversion. FCS techniques can offer a temporal resolution of tens of nanoseconds with resolving power down to single molecules, thus fluorescence correlation methods have great potential for investigating the dynamic structure–function mechanisms of IDPs/IDRs, particularly in connecting knowledge gained from molecular simulations and benchtop experiments. Furthermore, the configuration of confocal optical pathway and single-photon counting avalanche photodiode detector (SPAD), typically employed in FCS instruments, are identical to that used for alternating laser excitation (ALEX) or pulse interleaved excitation (PIE) smFRET experiments (Hellenkamp* et al.* 2018; Lerner* et al.* 2018, 2021). In fact, the same raw photon streaming data can be used for either ALEX/PIE smFRET analysis (“burst” analysis) and FCS study, simultaneously yielding nanometer structural (FRET) and nanosecond dynamic (FCS) information of the underlying molecular processes. The theoretical principles and experimental applications of the combined ALEX/PIE-smFRET and nanosecond FCS approach were elaborated by Schuler (Schuler 2018), and were subsequently demonstrated by a study about the IDRs of the SARS-CoV-2 nucleocapsid (N) protein (Cubuk* et al.* 2021). For the FRET labeled N-terminal (NTD), linker, or C-terminal domain within the intact N-protein, PIE-smFRET and FRET-FCS results revealed respectively a wide conformational distributions and nanosecond conformal dynamics that are consistent with properties of IDRs (Cubuk* et al.* 2021). Importantly, these experimental results can be collaborated with all-atom molecule simulation findings. Subsequent *in vitro* studies revealed N-proteins form liquid droplets in presence of RNA, leading to a proposal of viral genomic RNA packing by N-proteins through an LLPS mechanism (Cubuk* et al.* 2021). Another advantage of FCS techniques is the ability of determining molecular properties on the mesoscopic scale (*i.e.*, tens of thousands of molecules). Thus, our lab carried out a short duration (3 s) FCS experiment using a fluorescently labeled IDP, which was repeated hundreds of times. Thus, each 3-s experiment samples a mesoscopic population of IDP conformations (*e.g.*, hydrodynamic radii). The experimentally measured distributions of the IDP’s hydrodynamic radii are consistent with those obtained from smFRET studies and molecule simulation (unpublished results). Overall, the potential of the smFRET-FCS techniques for investigating dynamic structural mechanisms of IDPs/IDRs have yet to be fully explored.

## PERSPECTIVES

There are two major obstacles for the FCS techniques to gain wider applications in biomedical research in general and in LLPS research in particular: (1) relative obscurity of the principles of fluorescence correlation analysis; (2) lack of easy to operate FCS instrument and data analysis software. Thus, this review attempts to explain the theories of FACS and FCCS techniques in both intuitive and quantitative ways. Such explanation, preliminary in nature, services the purpose of emboldening potential users to employ the versatile FCS methods to solve their respective research questions. Biologist friendly FCS instrument also starts to emerge (*e.g.*, Fig. 6), aiming to solve the critical issue of automatic confocal pinhole alignment. Furthermore, FCS data analysis requires users to understand the basics of correlation analysis, as well as how fluorophore photophysics are intertwined with experimental data (*e.g.*, triplet dynamics). Therefore, an effective way to aid users is to develop software tools that enforce standardized and semi-automatic data analysis procedures. In LLPS research, there is increasing demand in understanding the quantitative molecular mechanisms of condensate formation, first *in vitro,* and then extending such understanding to live cells. The sensitivity, multifunctionality and quantitative nature of FCS methods are well suited to meet such research challenges. Particularly, the ultrasmall probing volume (*i.e.*, FCS volume element), single-molecule sensitivity and non-invasive optical approach make FCS one of the few techniques capable of live-cell experimentation. It shall be pointed out, fluorescence microscopy is primarily used to image subcellular structures: *i.e.*, even with the most advanced super-resolution techniques, mechanistic studies at molecular levels are still not possible in live cells. Yet, the same fluorescence photons used for microscopic imaging can also be used for spectroscopic analysis with ultimate single-molecule sensitivity. FCS techniques can be seamlessly integrated with a variety of microscope platforms: *e.g.*, confocal, multiphoton, TIRF, STED, light sheet, *etc*. Thus, not only subcellular structures can be imaged with increasingly fine resolution, but also molecular mechanisms supporting the subcellular structures can be investigated in live cells. This goal will drive future development and applications of FCS techniques.

## Conflict of interest

Zhulou Wang, Huizhi Zhang, Lin Jian, Bo Ding, Keying Huang, Wolun Zhang, Qian Xiao and Shaohui Huang declare that they have no conflict of interest.
